# Supplementary material for: A Critical View of the Application of the APEX Software (Aqueous Photochemistry of Environmentally-Occurring Xenobiotics) to Predict Photoreaction Kinetics in Surface Freshwaters
Source: Molecules. 2019 Dec 18;25(1):9. doi: 10.3390/molecules25010009 (PMC7017383; doi:10.3390/molecules25010009)
Supplement: Supplementary file 1 [file molecules-25-00009-s001.zip › 3.2.4_gcc-4.4.0.zip!/3.2.4_gcc-4.4.0/3.2.4_gcc-4.4.0/doc/octave/html/interpreter/Tests.html]

Tests - Untitled


Next: Models,
Previous: Statistical Plots,
Up: Statistics


---

### 25.4 Tests

Octave can perform several different statistical tests. The following
table summarizes the available tests.

|  |  |
| --- | --- |
| **Hypothesis** | **Test Functions** |
| Equal mean values | `anova`, `hotelling_test2`, `t_test_2`, `welch_test`, `wilcoxon_test`, `z_test_2` |
| Equal medians | `kruskal_wallis_test`, `sign_test` |
| Equal variances | `bartlett_test`, `manova`, `var_test` |
| Equal distributions | `chisquare_test_homogeneity`, `kolmogorov_smirnov_test_2`, `u_test` |
| Equal marginal frequencies | `mcnemar_test` |
| Equal success probabilities | `prop_test_2` |
| Independent observations | `chisquare_test_independence`, `run_test` |
| Uncorrelated observations | `cor_test` |
| Given mean value | `hotelling_test`, `t_test`, `z_test` |
| Observations from given distribution | `kolmogorov_smirnov_test` |
| Regression | `f_test_regression`, `t_test_regression` |

The tests return a p-value that describes the outcome of the test.
Assuming that the test hypothesis is true, the p-value is the probability
of obtaining a worse result than the observed one. So large p-values
corresponds to a successful test. Usually a test hypothesis is accepted
if the p-value exceeds 0.05.

— Function File: [pval, f, df\_b, df\_w] = **anova** (y, g)  
> Perform a one-way analysis of variance (ANOVA). The goal is to test
> whether the population means of data taken from k different
> groups are all equal.
>
> Data may be given in a single vector y with groups specified by
> a corresponding vector of group labels g (e.g., numbers from 1
> to k). This is the general form which does not impose any
> restriction on the number of data in each group or the group labels.
>
> If y is a matrix and g is omitted, each column of y
> is treated as a group. This form is only appropriate for balanced
> ANOVA in which the numbers of samples from each group are all equal.
>
> Under the null of constant means, the statistic f follows an F
> distribution with df\_b and df\_w degrees of freedom.
>
> The p-value (1 minus the CDF of this distribution at f) is
> returned in pval.
>
> If no output argument is given, the standard one-way ANOVA table is
> printed.

— Function File: [pval, chisq, df] = **bartlett\_test** (x1, ...)  
> Perform a Bartlett test for the homogeneity of variances in the data
> vectors x1, x2, ..., xk, where k > 1.
>
> Under the null of equal variances, the test statistic chisq
> approximately follows a chi-square distribution with df degrees of
> freedom.
>
> The p-value (1 minus the CDF of this distribution at chisq) is
> returned in pval.
>
> If no output argument is given, the p-value is displayed.

— Function File: [pval, chisq, df] = **chisquare\_test\_homogeneity** (x, y, c)  
> Given two samples x and y, perform a chisquare test for
> homogeneity of the null hypothesis that x and y come from
> the same distribution, based on the partition induced by the
> (strictly increasing) entries of c.
>
> For large samples, the test statistic chisq approximately follows a
> chisquare distribution with df = `length (`c`)`
> degrees of freedom.
>
> The p-value (1 minus the CDF of this distribution at chisq) is
> returned in pval.
>
> If no output argument is given, the p-value is displayed.

— Function File: [pval, chisq, df] = **chisquare\_test\_independence** (x)  
> Perform a chi-square test for independence based on the contingency
> table x. Under the null hypothesis of independence,
> chisq approximately has a chi-square distribution with
> df degrees of freedom.
>
> The p-value (1 minus the CDF of this distribution at chisq) of the
> test is returned in pval.
>
> If no output argument is given, the p-value is displayed.

— Function File: **cor\_test** (x, y, alt, method)  
> Test whether two samples x and y come from uncorrelated
> populations.
>
> The optional argument string alt describes the alternative
> hypothesis, and can be `"!="` or `"<>"` (non-zero),
> `">"` (greater than 0), or `"<"` (less than 0). The
> default is the two-sided case.
>
> The optional argument string method specifies on which
> correlation coefficient the test should be based. If method is
> `"pearson"` (default), the (usual) Pearson's product moment
> correlation coefficient is used. In this case, the data should come
> from a bivariate normal distribution. Otherwise, the other two
> methods offer nonparametric alternatives. If method is
> `"kendall"`, then Kendall's rank correlation tau is used. If
> method is `"spearman"`, then Spearman's rank correlation
> rho is used. Only the first character is necessary.
>
> The output is a structure with the following elements:
>
> pval: The p-value of the test. stat: The value of the test statistic. dist: The distribution of the test statistic. params: The parameters of the null distribution of the test statistic. alternative: The alternative hypothesis. method: The method used for testing.
>
> If no output argument is given, the p-value is displayed.

— Function File: [pval, f, df\_num, df\_den] = **f\_test\_regression** (y, x, rr, r)  
> Perform an F test for the null hypothesis rr \* b = r in a classical
> normal regression model y = X \* b + e.
>
> Under the null, the test statistic f follows an F distribution
> with df\_num and df\_den degrees of freedom.
>
> The p-value (1 minus the CDF of this distribution at f) is
> returned in pval.
>
> If not given explicitly, r = 0.
>
> If no output argument is given, the p-value is displayed.

— Function File: [pval, tsq] = **hotelling\_test** (x, m)  
> For a sample x from a multivariate normal distribution with unknown
> mean and covariance matrix, test the null hypothesis that `mean
> (`x`) ==` m.
>
> Hotelling's T^2 is returned in tsq. Under the null,
> (n-p) T^2 / (p(n-1)) has an F distribution with p and
> n-p degrees of freedom, where n and p are the
> numbers of samples and variables, respectively.
>
> The p-value of the test is returned in pval.
>
> If no output argument is given, the p-value of the test is displayed.

— Function File: [pval, tsq] = **hotelling\_test\_2** (x, y)  
> For two samples x from multivariate normal distributions with
> the same number of variables (columns), unknown means and unknown
> equal covariance matrices, test the null hypothesis `mean
> (`x`) == mean (`y`)`.
>
> Hotelling's two-sample T^2 is returned in tsq. Under the null,
>
> ```
>           (n_x+n_y-p-1) T^2 / (p(n_x+n_y-2))
> ```
>
> has an F distribution with p and n\_x+n\_y-p-1 degrees of
> freedom, where n\_x and n\_y are the sample sizes and
> p is the number of variables.
>
> The p-value of the test is returned in pval.
>
> If no output argument is given, the p-value of the test is displayed.

— Function File: [pval, ks] = **kolmogorov\_smirnov\_test** (x, dist, params, alt)  
> Perform a Kolmogorov-Smirnov test of the null hypothesis that the
> sample x comes from the (continuous) distribution dist. I.e.,
> if F and G are the CDFs corresponding to the sample and dist,
> respectively, then the null is that F == G.
>
> The optional argument params contains a list of parameters of
> dist. For example, to test whether a sample x comes from
> a uniform distribution on [2,4], use
>
> ```
>           kolmogorov_smirnov_test(x, "uniform", 2, 4)
> ```
>
> dist can be any string for which a function dist\_cdf
> that calculates the CDF of distribution dist exists.
>
> With the optional argument string alt, the alternative of
> interest can be selected. If alt is `"!="` or
> `"<>"`, the null is tested against the two-sided alternative F
> != G. In this case, the test statistic ks follows a two-sided
> Kolmogorov-Smirnov distribution. If alt is `">"`, the
> one-sided alternative F > G is considered. Similarly for `"<"`,
> the one-sided alternative F > G is considered. In this case, the
> test statistic ks has a one-sided Kolmogorov-Smirnov
> distribution. The default is the two-sided case.
>
> The p-value of the test is returned in pval.
>
> If no output argument is given, the p-value is displayed.

— Function File: [pval, ks, d] = **kolmogorov\_smirnov\_test\_2** (x, y, alt)  
> Perform a 2-sample Kolmogorov-Smirnov test of the null hypothesis
> that the samples x and y come from the same (continuous)
> distribution. I.e., if F and G are the CDFs corresponding to the
> x and y samples, respectively, then the null is that F ==
> G.
>
> With the optional argument string alt, the alternative of
> interest can be selected. If alt is `"!="` or
> `"<>"`, the null is tested against the two-sided alternative F
> != G. In this case, the test statistic ks follows a two-sided
> Kolmogorov-Smirnov distribution. If alt is `">"`, the
> one-sided alternative F > G is considered. Similarly for `"<"`,
> the one-sided alternative F < G is considered. In this case, the
> test statistic ks has a one-sided Kolmogorov-Smirnov
> distribution. The default is the two-sided case.
>
> The p-value of the test is returned in pval.
>
> The third returned value, d, is the test statistic, the maximum
> vertical distance between the two cumulative distribution functions.
>
> If no output argument is given, the p-value is displayed.

— Function File: [pval, k, df] = **kruskal\_wallis\_test** (x1, ...)  
> Perform a Kruskal-Wallis one-factor "analysis of variance".
>
> Suppose a variable is observed for k > 1 different groups, and
> let x1, ..., xk be the corresponding data vectors.
>
> Under the null hypothesis that the ranks in the pooled sample are not
> affected by the group memberships, the test statistic k is
> approximately chi-square with df = k - 1 degrees of
> freedom.
>
> If the data contains ties (some value appears more than once)
> k is divided by
>
> 1 - sum\_ties / (n^3 - n)
>
> where sum\_ties is the sum of t^2 - t over each group
> of ties where t is the number of ties in the group and n
> is the total number of values in the input data. For more info on
> this adjustment see "Use of Ranks in One-Criterion Variance Analysis"
> in Journal of the American Statistical Association, Vol. 47,
> No. 260 (Dec 1952) by William H. Kruskal and W. Allen Wallis.
>
> The p-value (1 minus the CDF of this distribution at k) is
> returned in pval.
>
> If no output argument is given, the p-value is displayed.

— Function File: **manova** (y, g)  
> Perform a one-way multivariate analysis of variance (MANOVA). The
> goal is to test whether the p-dimensional population means of data
> taken from k different groups are all equal. All data are
> assumed drawn independently from p-dimensional normal distributions
> with the same covariance matrix.
>
> The data matrix is given by y. As usual, rows are observations
> and columns are variables. The vector g specifies the
> corresponding group labels (e.g., numbers from 1 to k).
>
> The LR test statistic (Wilks' Lambda) and approximate p-values are
> computed and displayed.

— Function File: [pval, chisq, df] = **mcnemar\_test** (x)  
> For a square contingency table x of data cross-classified on
> the row and column variables, McNemar's test can be used for testing
> the null hypothesis of symmetry of the classification probabilities.
>
> Under the null, chisq is approximately distributed as chisquare
> with df degrees of freedom.
>
> The p-value (1 minus the CDF of this distribution at chisq) is
> returned in pval.
>
> If no output argument is given, the p-value of the test is displayed.

— Function File: [pval, z] = **prop\_test\_2** (x1, n1, x2, n2, alt)  
> If x1 and n1 are the counts of successes and trials in
> one sample, and x2 and n2 those in a second one, test the
> null hypothesis that the success probabilities p1 and p2
> are the same. Under the null, the test statistic z
> approximately follows a standard normal distribution.
>
> With the optional argument string alt, the alternative of
> interest can be selected. If alt is `"!="` or
> `"<>"`, the null is tested against the two-sided alternative
> p1 != p2. If alt is `">"`, the one-sided
> alternative p1 > p2 is used. Similarly for `"<"`,
> the one-sided alternative p1 < p2 is used.
> The default is the two-sided case.
>
> The p-value of the test is returned in pval.
>
> If no output argument is given, the p-value of the test is displayed.

— Function File: [pval, chisq] = **run\_test** (x)  
> Perform a chi-square test with 6 degrees of freedom based on the
> upward runs in the columns of x. Can be used to test whether
> x contains independent data.
>
> The p-value of the test is returned in pval.
>
> If no output argument is given, the p-value is displayed.

— Function File: [pval, b, n] = **sign\_test** (x, y, alt)  
> For two matched-pair samples x and y, perform a sign test
> of the null hypothesis PROB (x > y) == PROB (x <
> y) == 1/2. Under the null, the test statistic b roughly
> follows a binomial distribution with parameters n `= sum
> (`x `!=` y`)` and p = 1/2.
>
> With the optional argument `alt`, the alternative of interest
> can be selected. If alt is `"!="` or `"<>"`, the
> null hypothesis is tested against the two-sided alternative PROB
> (x < y) != 1/2. If alt is `">"`, the
> one-sided alternative PROB (x > y) > 1/2 ("x is
> stochastically greater than y") is considered. Similarly for
> `"<"`, the one-sided alternative PROB (x > y) < 1/2
> ("x is stochastically less than y") is considered. The default is
> the two-sided case.
>
> The p-value of the test is returned in pval.
>
> If no output argument is given, the p-value of the test is displayed.

— Function File: [pval, t, df] = **t\_test** (x, m, alt)  
> For a sample x from a normal distribution with unknown mean and
> variance, perform a t-test of the null hypothesis `mean
> (`x`) ==` m. Under the null, the test statistic t
> follows a Student distribution with df `= length (`x`)
> - 1` degrees of freedom.
>
> With the optional argument string alt, the alternative of
> interest can be selected. If alt is `"!="` or
> `"<>"`, the null is tested against the two-sided alternative
> `mean (`x`) !=` m. If alt is `">"`, the
> one-sided alternative `mean (`x`) >` m is considered.
> Similarly for "<", the one-sided alternative `mean
> (`x`) <` m is considered. The default is the two-sided
> case.
>
> The p-value of the test is returned in pval.
>
> If no output argument is given, the p-value of the test is displayed.

— Function File: [pval, t, df] = **t\_test\_2** (x, y, alt)  
> For two samples x and y from normal distributions with unknown means
> and unknown equal variances, perform a two-sample t-test of the null
> hypothesis of equal means. Under the null, the test statistic
> t follows a Student distribution with df degrees of
> freedom.
>
> With the optional argument string alt, the alternative of
> interest can be selected. If alt is `"!="` or
> `"<>"`, the null is tested against the two-sided alternative
> `mean (`x`) != mean (`y`)`. If alt is `">"`,
> the one-sided alternative `mean (`x`) > mean (`y`)` is
> used. Similarly for `"<"`, the one-sided alternative `mean
> (`x`) < mean (`y`)` is used. The default is the two-sided
> case.
>
> The p-value of the test is returned in pval.
>
> If no output argument is given, the p-value of the test is displayed.

— Function File: [pval, t, df] = **t\_test\_regression** (y, x, rr, r, alt)  
> Perform an t test for the null hypothesis rr `*` b `=` r in a classical normal regression model y `=` x `*` b `+` e. Under the null, the test statistic t
> follows a t distribution with df degrees of freedom.
>
> If r is omitted, a value of 0 is assumed.
>
> With the optional argument string alt, the alternative of
> interest can be selected. If alt is `"!="` or
> `"<>"`, the null is tested against the two-sided alternative
> rr `*` b `!=` r. If alt is `">"`, the
> one-sided alternative rr `*` b `>` r is used.
> Similarly for "<", the one-sided alternative rr `*` b `<` r is used. The default is the two-sided case.
>
> The p-value of the test is returned in pval.
>
> If no output argument is given, the p-value of the test is displayed.

— Function File: [pval, z] = **u\_test** (x, y, alt)  
> For two samples x and y, perform a Mann-Whitney U-test of
> the null hypothesis PROB (x > y) == 1/2 == PROB (x
> < y). Under the null, the test statistic z approximately
> follows a standard normal distribution. Note that this test is
> equivalent to the Wilcoxon rank-sum test.
>
> With the optional argument string alt, the alternative of
> interest can be selected. If alt is `"!="` or
> `"<>"`, the null is tested against the two-sided alternative
> PROB (x > y) != 1/2. If alt is `">"`, the
> one-sided alternative PROB (x > y) > 1/2 is considered.
> Similarly for `"<"`, the one-sided alternative PROB (x >
> y) < 1/2 is considered. The default is the two-sided case.
>
> The p-value of the test is returned in pval.
>
> If no output argument is given, the p-value of the test is displayed.

— Function File: [pval, f, df\_num, df\_den] = **var\_test** (x, y, alt)  
> For two samples x and y from normal distributions with
> unknown means and unknown variances, perform an F-test of the null
> hypothesis of equal variances. Under the null, the test statistic
> f follows an F-distribution with df\_num and df\_den
> degrees of freedom.
>
> With the optional argument string alt, the alternative of
> interest can be selected. If alt is `"!="` or
> `"<>"`, the null is tested against the two-sided alternative
> `var (`x`) != var (`y`)`. If alt is `">"`,
> the one-sided alternative `var (`x`) > var (`y`)` is
> used. Similarly for "<", the one-sided alternative `var
> (`x`) > var (`y`)` is used. The default is the two-sided
> case.
>
> The p-value of the test is returned in pval.
>
> If no output argument is given, the p-value of the test is displayed.

— Function File: [pval, t, df] = **welch\_test** (x, y, alt)  
> For two samples x and y from normal distributions with
> unknown means and unknown and not necessarily equal variances,
> perform a Welch test of the null hypothesis of equal means.
> Under the null, the test statistic t approximately follows a
> Student distribution with df degrees of freedom.
>
> With the optional argument string alt, the alternative of
> interest can be selected. If alt is `"!="` or
> `"<>"`, the null is tested against the two-sided alternative
> `mean (`x`) !=` m. If alt is `">"`, the
> one-sided alternative mean(x) > m is considered. Similarly for
> `"<"`, the one-sided alternative mean(x) < m is
> considered. The default is the two-sided case.
>
> The p-value of the test is returned in pval.
>
> If no output argument is given, the p-value of the test is displayed.

— Function File: [pval, z] = **wilcoxon\_test** (x, y, alt)  
> For two matched-pair sample vectors x and y, perform a
> Wilcoxon signed-rank test of the null hypothesis PROB (x >
> y) == 1/2. Under the null, the test statistic z
> approximately follows a standard normal distribution when n > 25.
>
> **Warning**: This function assumes a normal distribution for z
> and thus is invalid for n <= 25.
>
> With the optional argument string alt, the alternative of
> interest can be selected. If alt is `"!="` or
> `"<>"`, the null is tested against the two-sided alternative
> PROB (x > y) != 1/2. If alt is `">"`, the one-sided
> alternative PROB (x > y) > 1/2 is considered. Similarly
> for `"<"`, the one-sided alternative PROB (x > y) <
> 1/2 is considered. The default is the two-sided case.
>
> The p-value of the test is returned in pval.
>
> If no output argument is given, the p-value of the test is displayed.

— Function File: [pval, z] = **z\_test** (x, m, v, alt)  
> Perform a Z-test of the null hypothesis `mean (`x`) ==` m for a sample x from a normal distribution with unknown
> mean and known variance v. Under the null, the test statistic
> z follows a standard normal distribution.
>
> With the optional argument string alt, the alternative of
> interest can be selected. If alt is `"!="` or
> `"<>"`, the null is tested against the two-sided alternative
> `mean (`x`) !=` m. If alt is `">"`, the
> one-sided alternative `mean (`x`) >` m is considered.
> Similarly for `"<"`, the one-sided alternative `mean
> (`x`) <` m is considered. The default is the two-sided
> case.
>
> The p-value of the test is returned in pval.
>
> If no output argument is given, the p-value of the test is displayed
> along with some information.

— Function File: [pval, z] = **z\_test\_2** (x, y, v\_x, v\_y, alt)  
> For two samples x and y from normal distributions with
> unknown means and known variances v\_x and v\_y, perform a
> Z-test of the hypothesis of equal means. Under the null, the test
> statistic z follows a standard normal distribution.
>
> With the optional argument string alt, the alternative of
> interest can be selected. If alt is `"!="` or
> `"<>"`, the null is tested against the two-sided alternative
> `mean (`x`) != mean (`y`)`. If alt is `">"`, the
> one-sided alternative `mean (`x`) > mean (`y`)` is used.
> Similarly for `"<"`, the one-sided alternative `mean
> (`x`) < mean (`y`)` is used. The default is the two-sided
> case.
>
> The p-value of the test is returned in pval.
>
> If no output argument is given, the p-value of the test is displayed
> along with some information.
